# A Novel Prognostic Indicator for Immunotherapy Response: Lymphocyte-to-Albumin (LA) Ratio Predicts Survival in Metastatic NSCLC Patients

**DOI:** 10.3390/cancers16142512

**Published:** 2024-07-11

**Authors:** Sedat Yildirim, Akif Dogan, Goncagul Akdag, Eyyup Cavdar, Oguzcan Kinikoglu, Sila Oksuz, Hacer Sahika Yildiz, Aysun Kucukoz Uzun, Deniz Isik, Heves Surmeli, Tugba Basoglu, Ozlem Nuray Sever, Hatice Odabas, Mahmut Emre Yildirim, Nedim Turan

**Affiliations:** 1 Department of Medical Oncology, Kartal Dr. Lütfi Kirdar City Hospital, Health Science University, Istanbul 34865, Turkey; akif.dogan1@saglik.gov.tr (A.D.); akdaggoncagul@gmail.com (G.A.); ogokinikoglu@yahoo.com (O.K.); silaoksuz@gmail.com (S.O.); h.sahikayildiz@gmail.com (H.S.Y.); dnz.1984@yahoo.com (D.I.); hevessurmeli@hotmail.com (H.S.); basoglutugba@gmail.com (T.B.); ozlem.sever@hotmail.com (O.N.S.); odabashatice@yahoo.com (H.O.); emremahmutyildirim@gmail.com (M.E.Y.); turan.nedim@hotmail.com (N.T.); 2 Department of Medical Oncology, Faculty of Medicine, Tekirdag Namik Kemal University, Tekirdağ 59030, Turkey; eyyupcavdar@hotmail.com; 3 Department of Nuclear Medicine, Kartal Dr. Lütfi Kirdar City Hospital, Health Science University, Istanbul 34865, Turkey; aysunkucukoz@yahoo.com

**Keywords:** nivolumab, LA index, NSCLC, immunotherapy, prognostic factor, lung cancer

## Abstract

**Simple Summary:**

Based on this study, we define a new biomarker termed lymphocyte-to-albumin ratio (LAR) that may predict the prognosis for patients with metastatic NSCLC treated with immunotherapy. This study aims to determine the relationship of the LA index with patients’ survival rate through studying the records of 227 patients who were treated with nivolumab after one or multiple cycles of chemotherapy. Therefore, the results showed that a higher LA index is significantly related to better overall survival (OS) and progression-free survival (PFS). Overall, the LA index deserves its merit of being a simple, cost-effective, noninvasive method applicable in NSCLC immunotherapy as a clinically practical tool for predicting treatment outcomes.

**Abstract:**

Objective: Immunotherapies are commonly employed for the treatment of non-small-cell lung cancer (NSCLC). However, predictive biomarkers still need to be improved to predict responses to these agents. The lymphocyte–albumin (LA) laboratory index has not been evaluated before in this patient group. The aim of this study was to analyze the relation between the LA index and the survival rate of metastatic NSCLC patients who had immunotherapy after at least one round of chemotherapy. Methods: The research included 227 patients diagnosed with metastatic NSCLC, who were administered nivolumab after at least one round of chemotherapy. The LA index was calculated by multiplying lymphocyte count and albumin concentration. The optimal threshold values for the index were established by the examination of the ROC curve for both overall survival (OS) and progression-free survival (PFS). Oncological data were obtained retrospectively from patient files, and survival analyses were performed. Results: The median follow-up was 7.9 months. Progression was observed in 129 (56.9%) patients. A total of 97 (42.7%) patients died during the follow-up. The cutoff values of the LA index to predict OS and PFS were determined as 52.87 and 57.67, respectively. The low-LA group had significantly lowered OS and PFS compared to the high-LA group. LA was found to be an independent prognostic factor for PFS (hazard ratio 4.47; 95% confidence interval, 2.73–7.34; *p* < 0.001) and OS (hazard ratio 6.24; 95% confidence interval, 3.46–11.25; *p* < 0.001) in the multivariate regression analysis. Conclusions: In this study, we observed that the LA index independently predicts OS and PFS in immunotherapy-treated metastatic NSCLC patients. Its ease of application, low cost, and noninvasive nature make it a potential guide for clinicians in predicting treatment responses and survival.

## 1. Introduction

Lung cancer remains the leading cause of cancer-related deaths in both men and women despite remarkable advances in its treatment. Despite increased treatment options, five-year overall survival (OS) rates remain around 20% [1]. Non-small-cell lung cancer (NSCLC) is categorized into many subtypes, such as adenocarcinoma, squamous cell carcinoma, and large-cell carcinoma [2]. NSCLC constitutes 85–90% of all lung malignancies. In the last ten years, the role of immunotherapy in treating NSCLC has become increasingly evident and the mainstay of treatment [3]. Immune checkpoint inhibitors, including atezolizumab, nivolumab, and pembrolizumab, have shown improvements in terms of progression-free survival (PFS) and overall survival (OS) in the treatment of NSCLC [4,5,6].

Following the demonstration of PFS and OS benefits of immunotherapy in lung cancer, numerous studies have been conducted to identify predictive and prognostic biomarkers to predict this benefit [4,5,6,7]. To this end, tumor-infiltrating immune cells, gene-expression-based biomarkers, PD-L1 expression level, tumor mutational burden, complete blood count, peripheral blood mononuclear cells, extracellular structural agents, microbiota, and imaging-based biomarkers have been investigated [8,9]. However, the investigated biomarkers have various limitations, and the search for an ideal biomarker continues [7,8,9,10].

There is increasing evidence from research indicating that inflammation and nutritional condition play a significant role in the initiation, advancement, and metastasis of cancer [11]. Neutrophils, monocyte-derived macrophages, and platelets, which contribute to the tumor microenvironment, are negative prognostic indicators, while tumor-infiltrating lymphocytes are positive prognostic indicators [12,13]. Albumin, a biomarker found in the peripheral blood, is one of the indicators of inflammation and nutritional status, such as weight loss, sarcopenia, and hypercatabolic syndrome [14,15]. Other laboratory indices that are derived from peripheral blood cells and proteins, such as the neutrophil-to-lymphocyte ratio (NLR), monocyte-to-lymphocyte ratio (MLR), systemic immune-inflammation index (SII), platelet-to-lymphocyte ratio (PLR), and monocyte-to-albumin ratio (MAR), have shown prognostic significance in several solid organ cancers [12,16,17,18,19]. The LA index, obtained by multiplying the lymphocyte count and albumin concentration, was first reported to be a reliable new prognostic marker in stage II and III rectal cancer [20]. The prognostic significance of LA as a biomarker has not been previously evaluated in any patient group with cancer who received immunotherapy. The objective of this research was to examine the predictive significance of LA in 227 patients with advanced NSCLC who had immunotherapy in the second-and-beyond line of therapy.

## 2. Materials and Methods

Study population: This research included 227 individuals diagnosed with NSCLC admitted to our center between May 2021 and April 2023 who received nivolumab in the second line and beyond. The inclusion criteria for this study were individuals aged 18 years or older, diagnosed with NSCLC and receiving therapy with nivolumab. Patients had initially received standard first-line chemotherapy prior to nivolumab administration. Patients with early-stage disease, who did not receive nivolumab treatment, who had a hematological malignancy, who had a concurrent active solid malignancy, who had not been evaluated for treatment response, or who had missing data were not included in the research.

Data collection. The demographic data, treatment doses, durations, treatment responses, and tumor characteristics of all patients included were obtained by retrospective review of medical oncology files. The distribution of the sociodemographic and clinical characteristics of the patients is shown in Table 1. The American Joint Committee on Cancer (AJCC) Staging Manual was used to assess each patient’s tumor stage. An expert radiologist assessed the patients’ responses to nivolumab according to the immune-related positron emission tomography/computed tomography response criteria in solid tumors (iRECIST) [21]. Before receiving nivolumab, 149 (65.6%) of the 227 patients had one line of chemotherapy, 57 (25.1%) had two lines of chemotherapy, and 21 (9.3%) had three or more lines of chemotherapy (Table 1). Detailed data were collected on patients’ age, gender, ECOG performance status, histopathological subtype of NSCLC, initial stage at diagnosis, number of lines of chemotherapy received before nivolumab, initial radiological response to nivolumab, and progression status. Survival status and follow-up duration were also recorded. Laboratory values for calculating various indices, including the LA index, were collected from peripheral blood samples taken within two weeks before the initiation of nivolumab treatment.

OS and PFS Measurement: Overall survival (OS) was determined by measuring the duration from the initiation of nivolumab medication to the occurrence of death (in months), or until the most recent follow-up for patients who were still alive. Progression-free survival (PFS) was determined by measuring the duration from the initiation of nivolumab therapy to the occurrence of disease progression or death, whichever happened first. The period was measured in months. For both OS and PFS measurements, the time from the start of first-line chemotherapy to these events was also recorded to provide a comprehensive evaluation of treatment impact.

Laboratory values and calculations: The LA index was calculated by multiplying the peripheral blood lymphocyte count (cells/μL) by the serum albumin concentration (g/dL). The lymphocyte count and albumin concentration were measured using standard automated laboratory techniques. Lymphocyte counts were obtained from a complete blood count (CBC) test, and albumin levels were determined through a biochemical analysis of the blood samples.

Cutoff values calculation: The optimal cutoff values for the laboratory indices, including the LA index, were determined by receiver operating characteristic (ROC) curve analysis. The ROC curve was plotted to assess the sensitivity and specificity of each index in predicting overall survival (OS) and progression-free survival (PFS). The Youden Index (sensitivity + specificity − 1) was used to identify the optimal cutoff point, which maximized the sum of sensitivity and specificity. These cutoff values were then used to stratify patients into different risk groups for further survival analysis.

### 2.1. Outcomes

OS was determined by measuring the duration from the initiation of nivolumab medication to the occurrence of death (in months), or until the most recent follow-up for patients who were still alive. PFS was determined by measuring the duration from the initiation of nivolumab therapy to the occurrence of disease progression or death, whichever happened first. The period was measured in months.

### 2.2. Statistical Analysis

The statistical analyses were conducted using IBM SPSS Statistics for Windows, version 25.0, which is a software package developed by IBM Corp. in Armonk, NY, USA. Descriptive statistics were reported as n and %, whereas the categorical variables were provided as the mean ± SD or median (min–max). The results of the ROC curve analysis for predicting mortality and progression by various numerical parameter scores are presented. The Kaplan–Meier method compared OS and PFS times between groups formed according to patient and tumor characteristics. Finally, the Multivariable Cox Regression analysis results of various clinical factors on OS and PFS are presented. A statistically significant *p*-value was defined as less than 0.05.

## 3. Results

The median age of the patients in the study was 63 (range 24–88) years, and 102 patients (44.9%) were aged 65 years or older. Thirty-one patients (13.7%) were female. Eighty-nine patients (39.2%) had squamous cell carcinoma. Before receiving nivolumab, 149 (65.6%) of the 227 patients had one line of chemotherapy, 57 (25.1%) had two lines of chemotherapy, and 21 (9.3%) had three or more lines of chemotherapy.

The median follow-up time was 20 months. During the follow-up period, 97 (42.7%) patients died, and 89 (39.2%) progressed. In evaluating treatment response in 227 patients who received nivolumab, 12 patients (5.3%) had a complete response (pCR). Table 1 shows the patients’ sociodemographic, clinical, and tumor characteristics.

**Figure 1:**
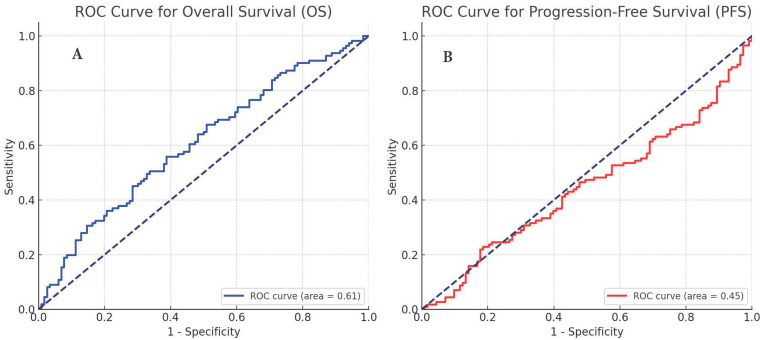
ROC Curve for of LA index, overall survival (**A**), and progression-free survival (**B**).

Tables S1 and S2 give the cutoff values of the laboratory indices calculated before treatment for OS and PFS. The ROC curve for the LA index for overall survival (A) and progression-free survival (B) is shown in Figure 1. Figure 1A demonstrates the ROC curve of the LA index for OS, which confirms the program’s ability to differentiate individuals with varying survival rates. The area under the curve (AUC) indicates the level of accuracy, with higher AUC values implying a stronger predicting ability and lower difficulty. Progression-free Survival (PFS) is shown in Figure 1B, which illustrates the same approach that forecasts the amount of time before illness progression.

**Figure 2:**
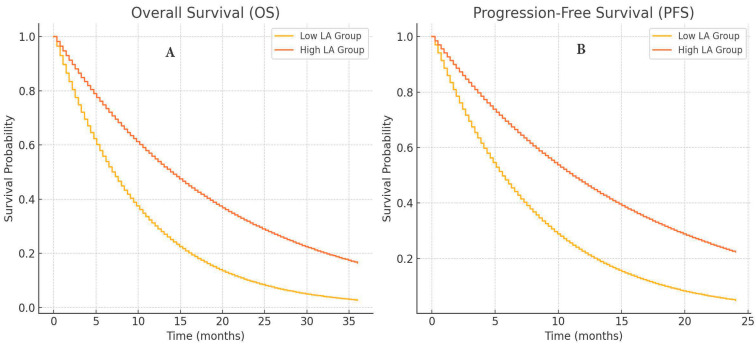
Survival outcomes according to LA index, overall survival (**A**), and progression-free survival (**B**).

Figure 2 shows survival outcomes according to the LA index for the low-LA group and high-LA group. Figure 2A represents the Kaplan–Meier overall survival (OS) curve of patients in low- as well as high-LA groups. The results also revealed a favorable patient survival probability for patients in the high-LA group compared to those in the low-LA group, as confirmed by the statistically significant difference in Kaplan–Meier survival curves. Figure 2B illustrates the PFS results in the same groups. As in previous studies, the high-LA group had better survival probabilities, suggesting that the LA index is a good prognostic indicator for this population of patients [12,20].

The median OS was 15.6 months. The low-LA-group’s median OS was 7 months, but the high-LA-group’s median OS was not reached (*p* ≤ 0.001). In the univariate analysis for OS, the ECOG score, LA index, NLR, PLR, MLR, SII, and MAR (*p* ≤ 0.001; *p* ≤ 0.001; *p* ≤ 0.001; *p* ≤ 0.001; *p* ≤ 0.001; *p* ≤ 0.001; *p* = 0.011, respectively) were statistically significant, but in the multivariate analysis, only the ECOG score, LA index, SII, and MAR indices were statistically significant (*p* = 0.003; *p* ≤ 0.001; *p* = 0.045; *p* = 0.006, respectively, Table 2).

The median PFS was 8 months. The low-LA-group’s median PFS was 4.8 months, but the high-LA-group’s median PFS was not reached (*p* ≤ 0.001). The multivariate analysis revealed that only the ECOG score, LA index, SII, and MAR indices were statistically significantly prognostic (*p* = 0.009, *p* ≤ 0.001, *p* = 0.032; *p* = 0.002, respectively), but in the univariate analysis for PFS, the ECOG score, LA index, NLR, PLR, SII, and MAR (*p* ≤ 0.001, *p* ≤ 0.001; *p* ≤ 0.001; *p* ≤ 0.001, *p* = 0.001, *p* = 0.003, respectively) were statistically significantly related (Table 3).

## 4. Discussion

Many studies demonstrate the relationship between inflammation and nutrition in cancer patients and survival. Peripheral laboratory indices are valuable in this context as they can easily reveal this relationship. This study demonstrates that the LA index, a laboratory indicator not previously documented in the literature as being linked to survival in patients with NSCLC, is an independent prognostic factor for PFS and OS. We have strengthened our study by analyzing the LA index and other indices associated with survival in these patients.

There is a strong relationship between inflammation, nutrition, and survival in lung cancer. Peripheral blood values can generally reflect nutritional status and inflammation levels [22]. The first parameter of the LA index, the peripheral blood lymphocyte count, reflects CD8+T lymphocytes, which are markers of the body’s endogenous anti-tumor capacity [23]. Cytotoxic CD8+T lymphocytes that target tumor antigens are essential for cancer immunotherapy since they induce apoptosis in cancer cells [24]. Furthermore, increased tumor-infiltrating-lymphocyte numbers have been associated with improved NSCLC survival [25]. The other parameter of the LA index, albumin, is an acute-phase reactant negatively correlated with inflammation. In addition, low albumin levels are one of the indicators of cancer-associated malnutrition and can be an important indicator of poor prognosis [12]. An association was found between the degree of inflammation and the counts of neutrophils, platelets, and monocytes, indicating a positive connection. Conversely, a negative correlation was detected between the amount of inflammation and the lymphocyte count and the level of albumin [26].

The literature describes the relationship between laboratory indices such as the NLR, MLR, PLR, SII, and MAR, which reflect inflammation and immunity in peripheral blood, and survival. This relationship is consistent with our study [14,15,16,17,18,19,20,26]. Our findings demonstrate that the LA index possesses better predictive capabilities compared with the NLR, PLR, MLR, SII, and MAR. Compared to inflammatory-response-orientated indices like NLR and PLR, the LA index involves nutritional status, which enhances the understanding of the patient’s state. These two reflections of inflammation and nutrition may provide reasons why the statistical and clinical strengths of the LA index are greater for predicting OS and PFS. Further, they noted that the LA index is easy to compute and can be easily implemented as part of everyday clinical management without heavy instruments and processes.

The LA index, the primary focus of our study, was first defined in a study including 448 patients diagnosed with stage 2 and 3 colorectal cancer who received curative resection. The research demonstrated that a low LA value was an independent poor prognostic factor for overall survival (OS) and recurrence-free survival (RFS) [12,20]. In a current study performed by Wang et al., it was shown that LA serves as an independent prognostic marker for predicting disease-free survival (DFS) in breast cancer patients who undergo neoadjuvant chemotherapy [27,28]. A study of 216 patients with lung cancer between stage 1A and stage 3A, and 184 healthy individuals showed that the LA index is an important index for both diagnostic value and predicting early progression of the disease [29]. In addition to the literature, we showed the value of the LA index in metastatic NSCLC patients receiving immunotherapy.

The limitations of our investigation include its retrospective methodology, patients receiving immunotherapy second line or later, being a single-center study, and having a small sample size. However, its power is that it is the first time the LA index has been used in any cancer patient group receiving immunotherapy.

## 5. Conclusions

The LA index is a new, powerful prognostic laboratory index formed by the combination of peripheral-blood-derived lymphocytes and albumin, and it indirectly reflects nutritional status and inflammation. Our research is significant as it is the first to demonstrate the correlation between survival and the LA index in patients with metastatic NSCLC receiving immunotherapy. When considering the different number of lines of chemotherapy, the prognostic association of the LA index remained significant, indicating that the index is a robust predictor regardless of prior chemotherapy treatments. There is a need for quick and easily accessible biomarkers that predict survival in daily practice. In addition, the specificity and advantages of the LA index over other indices such as the NLR, PLR, MLR, SII, and MAR make it a superior prognostic tool. The results of our study should be strengthened with prospective and further studies.

## Figures and Tables

**Table 1:** Baseline clinical and demographic findings for the whole cohort.

Variables	N (227)	(%)
Age (years)		
Median (min–max)	63 (24–88)	
<65	125	55.1
≥65	102	44.9
Gender		
Female	31	13.7
Male	196	86.3
ECOG score		
0	161	70.9
1	49	21.6
2	17	7.5
Histopathology		
Squamous	89	39.2
Non-squamous	138	60.8
Initial stage		
Early stage at diagnosis	102	44.9
Metastatic at diagnosis	125	55.1
Number of lines of chemotherapy received before nivolumab		
1	149	65.6
2	57	25.1
3 or more	21	9.3
Initial radiological response evaluation to nivolumab		
Complete Response	12	5.3
Partial response	69	30.4
Stable disease	56	24.7
Confirmed progression	89	39.2
Pseudoprogression	1	0.4
Progression		
No	98	43.2
Yes	129	56.8
Status		
Alive	130	57.3
Exitus	97	42.7
Average Follow-Up Time	9.35 ± 5.82	

ECOG: Eastern Cooperative Oncology Group.

**Table 2:** Overall survival comparison of patients.

	Overall Survival						
			Univariate		Multivariate		
		n	1-Year OS (%)	*p*	HR	%95 CI	*p*
Age	<65	125	59.0	0.902			
	≥65	102	55.2				
Sex	Woman	31	51.7	0.878			
	Man	196	58.5				
ECOG score	0	161	58.0	<0.001	ref		0.011
	1	49	68.0		1.08	0.65–1.79	0.755
	2	17	0.0		2.80	1.43–5.50	0.003
Histopathology	Squamous	89	57.3	0.653			
	Non-squamous	138	57.6				
Number of lines of CT received before nivolumab	1	149	53.7	0.210			
	2	57	66.8				
	≥3	21	55.0				
LA	>52.87	122	82.0	<0.001	ref		
	≤52.87	105	29.8		6.24	3.46–11.25	<0.001
NLR	<3.61	118	73.0	<0.001	ref		
	≥3.61	109	39.5		0.97	0.55–1.70	0.923
MLR	<0.48	113	71.2	<0.001	ref		
	≥0.48	114	43.8		0.76	0.42–1.36	0.359
PLR	<196.48	105	70.7	<0.001	ref		
	≥196.48	122	43.0		0.89	0.53–1.49	0.669
SII	<921.57	118	70.6	<0.001	ref		
	≥921.57	109	43.2		1.75	1.01–3.03	0.045
MAR	<0.017	99	62.7	0.011	ref		
	≥0.017	128	54.0		2.07	1.22–3.51	0.006

Eastern Cooperative Oncology Group (ECOG), chemotherapy (CT), lymphocyte count and albumin concentration product (LA), neutrophil–lymphocyte ratio (NLR), monocyte–lymphocyte ratio (MLR), systemic immune-inflammation index (SII), platelet–lymphocyte ratio (PLR), monocyte–albumin ratio (MAR).

**Table 3:** Progression-free survival comparison of patients.

		PFS					
		Univariate			Multivariate		
		n	1-Year PFS (%)	*p*	HR	%95 CI	*p*
Age	<65	125	39.5	0.885			
	≥65	102	36.8				
Sex	Woman	31	35.2	0.572			
	Man	196	39.0				
ECOG score	0	161	36.7	<0.001	ref		0.022
	1	49	52.5		0.88	0.56–1.37	0.581
	2	17	0.0		2.27	1.22–4.21	0.009
Histopathology	Squamous	89	35.2	0.868			
	Non-squamous	138	40.4				
Number of lines of CT received before nivolumab	1	149	36.5	0.581			
	2	57	44.0				
	≥3	21	34.4				
LA	>57.67	106	65.2	<0.001	ref		
	≤57.67	121	15.4		4.47	2.73–7.34	<0.001
NLR	<3.35	109	51.6	<0.001	ref		
	≥3.35	118	26.5		0.79	0.48–1.30	0.356
MLR	<0.46	105	54.2	<0.001	ref		
	≥0.46	122	24.9		0.84	0.51–1.39	0.507
PLR	<190.30	110	50.2	0.001	ref		
	≥190.30	117	27.5		1.00	0.64–1.57	0.971
SII	<876.85	110	50.3	<0.001	ref		
	≥876.85	117	27.7		1.68	1.04–2.71	0.032
MAR	<0.016	87	49.2	0.003	ref		
	≥0.016	140	31.3		2.03	1.30–3.16	0.002

Eastern Cooperative Oncology Group (ECOG), chemotherapy (CT), lymphocyte count and albumin concentration product (LA), neutrophil–lymphocyte ratio (NLR), monocyte–lymphocyte ratio (MLR), systemic immune-inflammation index (SII), platelet–lymphocyte ratio (PLR), monocyte–albumin ratio (MAR).

## Data Availability

Although not publicly available, the datasets created and/or analyzed during the current study are available from the corresponding author upon justifiable request.

## Supplementary Materials

The following supporting information can be downloaded at: https://www.mdpi.com/article/10.3390/cancers16142512/s1, Table S1: Analysis of Predictive Values of Index Values in Distinguishing Mortality; Table S2: Analysis of Predictive Values of Index Values in Distinguishing Progression.

## References

[1] Siegel R.L., Miller K.D., Wagle N.S., Jemal A. (2023). Cancer statistics, 2023. CA Cancer J. Clin..

[2] WHO, Classification of Tumours Editorial Board (2021). Thoracic Tumours. WHO Classification of Tumours.

[3] Herbst R.S., Morgensztern D., Boshoff C. (2018). The biology and management of non-small cell lung cancer. Nature.

[4] Borghaei H., Gettinger S., Vokes E.E., Chow L.Q.M., Burgio M.A., de Castro Carpeno J., Pluzanski A., Arrieta O., Frontera O.A., Chiari R. (2021). Five-Year Outcomes from the Randomized, Phase III Trials CheckMate 017 and 057: Nivolumab versus Docetaxel in Previously Treated Non-Small-Cell Lung Cancer. J. Clin. Oncol..

[5] Herbst R.S., Garon E.B., Kim D.W., Cho B.C., Gervais R., Perez-Gracia J.L., Han J.Y., Majem M., Forster M.D., Monnet I. (2021). Five Year Survival Update From KEYNOTE-010: Pembrolizumab Versus Docetaxel for Previously Treated, Programmed Death-Ligand 1-Positive Advanced NSCLC. J. Thorac. Oncol..

[6] Mazieres J., Rittmeyer A., Gadgeel S., Hida T., Gandara D.R., Cortinovis D.L., Barlesi F., Yu W., Matheny C., Ballinger M. (2021). Atezolizumab versus Docetaxel in Pretreated Patients with NSCLC: Final Results from the Randomized Phase 2 POPLAR and Phase 3 OAK Clinical Trials. J. Thorac. Oncol..

[7] Sankar K., Ye J.C., Li Z., Zheng L., Song W., Hu-Lieskovan S. (2022). The role of biomarkers in personalized immunotherapy. Biomark Res..

[8] Tostes K., Siqueira A.P., Reis R.M., Leal L.F., Arantes L.M.R.B. (2023). Biomarkers for Immune Checkpoint Inhibitor Response in NSCLC: Current Developments and Applicability. Int. J. Mol. Sci..

[9] Shirasawa M., Yoshida T., Ohe Y. (2024). Biomarkers of immunotherapy for non-small cell lung cancer. Jpn. J. Clin. Oncol..

[10] Pabst L., Lopes S., Bertrand B., Creusot Q., Kotovskaya M., Pencreach E., Beau-Faller M., Mascaux C. (2023). Prognostic and Predictive Biomarkers in the Era of Immunotherapy for Lung Cancer. Int. J. Mol. Sci..

[11] Zitvogel L., Pietrocola F., Kroemer G. (2017). Nutrition, inflammation and cancer. Nat. Immunol..

[12] Yamamoto T., Kawada K., Obama K. (2021). Inflammation-Related Biomarkers for the Prediction of Prognosis in Colorectal Cancer Patients. Int. J. Mol. Sci..

[13] Tan Z., Xue H., Sun Y., Zhang C., Song Y., Qi Y. (2021). The Role of Tumor Inflammatory Microenvironment in Lung Cancer. Front. Pharmacol..

[14] Shi T., Wang Y., Peng Y., Wang M., Zhou Y., Gu W., Li Y., Zou J., Zhu N., Chen L. (2023). Advanced lung cancer inflammation index combined with geriatric nutritional risk index predict all-cause mortality in heart failure patients. BMC Cardiovasc. Disord..

[15] Zhang C.L., Gao M.Q., Jiang X.C., Pan X., Zhang X.Y., Li Y., Shen Q., Chen Y., Pang B. (2023). Research progress and value of albumin-related inflammatory markers in the prognosis of non-small cell lung cancer: A review of clinical evidence. Ann. Med..

[16] Zhao S.T., Chen X.X., Yang X.M., He S.C., Qian F.H. (2023). Application of Monocyte-to-Albumin Ratio and Neutrophil Percentage-to-Hemoglobin Ratio on Distinguishing Non-Small Cell Lung Cancer Patients from Healthy Subjects. Int. J. Gen. Med..

[17] Huai Q., Luo C., Song P., Bie F., Bai G., Li Y., Liu Y., Chen X., Zhou B., Sun X. (2023). Peripheral blood inflammatory biomarkers dynamics reflect treatment response and predict prognosis in non-small cell lung cancer patients with neoadjuvant immunotherapy. Cancer Sci..

[18] Savioli F., Morrow E.S., Dolan R.D., Romics L., Lannigan A., Edwards J., McMillan D.C. (2022). Prognostic role of preoperative circulating systemic inflammatory response markers in primary breast cancer: Meta-analysis. Br. J. Surg..

[19] Portale G., Bartolotta P., Azzolina D., Gregori D., Fiscon V. (2023). Prognostic role of platelet-to-lymphocyte ratio, neutrophil-to-lymphocyte, and lymphocyte-to-monocyte ratio in operated rectal cancer patients: Systematic review and meta-analysis. Langenbecks Arch. Surg..

[20] Yamamoto T., Kawada K., Hida K., Matsusue R., Itatani Y., Mizuno R., Yamaguchi T., Ikai I., Sakai Y. (2021). Combination of lymphocyte count and albumin concentration as a new prognostic biomarker for rectal cancer. Sci. Rep..

[21] David S.E., Mckinney K., Oken M., Lee H.K., Crumbaker M., Beg M.S., Holland J., American Society of Clinical Oncology (2024). NCCN Clinical Practice Guidelines in Oncology: Non-Small Cell Lung Cancer. Version 2. (NCCN.org., 2024).

[22] Matsuura S., Serizawa S., Yamashita R., Morikawa K., Ito Y., Hiramatsu T., Mochizuki E., Tanaka K., Akiyama N., Tsukui M. (2023). The Prognostic Nutritional Index before durvalumab after chemoradiation predict the overall survival in patients with stage III non-small cell lung cancer. Ann. Med..

[23] Manjarrez-Orduño N., Menard L.C., Kansal S., Fischer P., Kakrecha B., Jiang C., Cunningham M., Greenawalt D., Patel V., Yang M. (2018). Circulating T Cell Subpopulations Correlate with Immune Responses at the Tumor Site and Clinical Response to PD1 Inhibition in Non-Small Cell Lung Cancer. Front. Immunol..

[24] Chen S.C., Wu P.C., Wang C.Y., Kuo P.L. (2020). Evaluation of cytotoxic T lymphocyte-mediated anticancer response against tumor interstitium-simulating physical barriers. Sci. Rep..

[25] Zheng Q.M., Li Y.Y., Wang Y.P., Li G.X., Zhao M.M., Sun Z.G. (2023). Association between CD8+ tumor-infiltrating lymphocytes and prognosis of non-small cell lung cancer patients treated with PD-1/PD-L1 inhibitors: A systematic review and meta-analysis. Expert Rev. Anticancer Ther..

[26] Wang Y., Li Y., Chen P., Xu W., Wu Y., Che G. (2019). Prognostic value of the pretreatment systemic immune-inflammation index (SII) in patients with non-small cell lung cancer: A meta-analysis. Ann. Transl. Med..

[27] Wang M.D., Duan F.F., Hua X., Cao L., Xia W., Chen J.Y. (2023). A Novel Albumin-Related Nutrition Biomarker Predicts Breast Cancer Prognosis in Neoadjuvant Chemotherapy: A Two-Center Cohort Study. Nutrients.

[28] Heppner B., Untch M., Denkert C., Pfitzner B., Lederer B., Schmitt W., Eidtmann H., Fasching P., Tesch H., Solbach C. (2016). Tumor-Infiltrating Lymphocytes: A Predictive and Prognostic Biomarker in Neoadjuvant-Treated HER2-Positive Breast Cancer. Clin. Cancer Res..

[29] Chen X.X., Zhao S.T., Yang X.M., He S.C., Qian F.H. (2023). Additional diagnostic value of the monocyte to red blood cell count ratio and the product of lymphocyte count and albumin concentration in lung cancer management. Oncol. Lett..

