# Supplementary material for: A Novel Prognostic Indicator for Immunotherapy Response: Lymphocyte-to-Albumin (LA) Ratio Predicts Survival in Metastatic NSCLC Patients
Source: Cancers (Basel). 2024 Jul 11;16(14):2512. doi: 10.3390/cancers16142512 (PMC11274503; doi:10.3390/cancers16142512)
Supplement: Supplementary file 1 [file cancers-16-02512-s001.zip › cancers-3049273-supplementary.pdf]

A Novel Prognostic Indicator for Immunotherapy Response: Lymphocyte-to-Albumin (LA) Ratio Predicts Survival in Metastatic NSCLC Patients

Sedat Yildirim [1,*], Akif Dogan [1], Goncagul Akdag [1], Eyyup Cavdar [2], Oguzcan Kinikoglu [1], Sila Oksuz [1], Hacer Sahika Yildiz [1], Aysun Kucukoz Uzun [3], Deniz Isik [1], Heves Surmeli [1], Tugba Basoglu [1], Ozlem Nuray Sever [1], Hatice Odabas [1], Mahmut Emre Yildirim [1] and Nedim Turan [1]

[1] Department of Medical Oncology, Kartal Dr. Lütfi Kirdar City Hospital, Health Science University, Istanbul 34865, Turkey; akif.dogan1@saglik.gov.tr (A.D.); akdaggoncagul@gmail.com (G.A.); ogokinikoglu@yahoo.com (O.K.); silaoksuz@gmail.com (S.O.); h.sahikayildiz@gmail.com (H.S.Y.); dnz.1984@yahoo.com (D.I.); hevessurmeli@hotmail.com (H.S.); basoglutugba@gmail.com (T.B.); ozlem.sever@hotmail.com (O.N.S.); odabashatice@yahoo.com (H.O.); emremahmutyildirim@gmail.com (M.E.Y.); turan.nedim@hotmail.com (N.T.)

[2] Department of Medical Oncology, Faculty of Medicine, Tekirdag Namik Kemal University, Tekirdağ 59030, Turkey; eyyupcavdar@hotmail.com

[3] Department of Nuclear Medicine, Kartal Dr. Lütfi Kirdar City Hospital, Health Science University, Istanbul 34865, Turkey; aysunkucukoz@yahoo.com

* Correspondence: rezansedat@hotmail.com; Tel.: +90-05327904791

Table S1: Analysis of Predictive Values of Index Values in Distinguishing Mortality

Variables	AUC	%95 CI	Cut-off	Sensitivity (%)	Specificity (%)	p
LA	0.786	0.725-0.847	≤52.87	75.3	75.4	**<0.001**
NLR	0.721	0.655-0.788	≥3.61	63.9	63.8	**<0.001**
MLR	0.704	0.636-0.772	≥0.48	64.9	64.6	**<0.001**
PLR	0.681	0.611-0.751	≥196.48	60.8	60.8	**<0.001**
SII	0.680	0.610-0.750	≥921.57	62.9	63.1	**<0.001**
MAR	0.587	0.513-0.662	≥0.017	56.7	56.9	**0.024**

AUC, Area Under Curve; %95CI, Confidential Interval,), lymphocyte count and albumin concentration product (LA), neutrophil-lymphocyte ratio (NLR), monocyte-lymphocyte ratio (MLR), systemic immune-inflammation index (SII), platelet-lymphocyte ratio (PLR), monocyte albumin ratio (MAR)

Table S2: Analysis of Predictive Values of Index Values in Distinguishing Progression

Variables	AUC	%95 CI	Cut-off	Sensitivity (%)	Specificity (%)	p
LA	0.765	0.703-0.827	≤57.67	72.9	72.4	**<0.001**
NLR	0.723	0.658-0.788	≥3.35	63.6	63.3	**<0.001**
MLR	0.705	0.638-0.773	≥0.46	63.6	63.3	**<0.001**
PLR	0.685	0.616-0.753	≥190.30	59.7	59.2	**<0.001**
SII	0.685	0.617-0.753	≥876.85	61.2	61.2	**<0.001**
MAR	0.592	0.518-0.667	≥0.016	57.4	57.1	**0.017**

AUC, Area Under Curve; %95CI, Confidential Interval,), lymphocyte count and albumin concentration product (LA), neutrophil-lymphocyte ratio (NLR), monocyte-lymphocyte ratio (MLR), systemic immune-inflammation index (SII), platelet-lymphocyte ratio (PLR), monocyte albumin ratio (MAR)
